# Development and Characterization of Pullulan-Based Orodispersible Films of Iron

**DOI:** 10.3390/pharmaceutics15031027

**Published:** 2023-03-22

**Authors:** Maram Suresh Gupta, Tegginamath Pramod Kumar, Dinesh Reddy, Kamla Pathak, Devegowda Vishakante Gowda, A. V. Naresh Babu, Alhussain H. Aodah, El-Sayed Khafagy, Hadil Faris Alotaibi, Amr Selim Abu Lila, Afrasim Moin, Talib Hussin

**Affiliations:** 1 Department of Pharmaceutics, JSS College of Pharmacy, JSS Academy of Higher Education and Research, Mysore 570015, India; 2 Aavishkar Oral Strips Private Limited, Plot No. 109/3, Phase-II, Sector 2, Lane No. 6 IDA Cherlapally, Hyderabad 500051, India; 3 Pharmacy College Saifai, Uttar Pradesh University of Medical Sciences, Etawah 206130, India; 4 Department of Pharmaceutics, Cauvery College of Pharmacy, Mysuru 570028, India; 5 Department of Pharmaceutics, College of Pharmacy, Prince Sattam Bin Abdulaziz University, Al-kharj 11942, Saudi Arabia; 6 Department of Pharmaceutics and Industrial Pharmacy, Faculty of Pharmacy, Suez Canal University, Ismailia 41522, Egypt; 7 Department of Pharmaceutical Sciences, College of Pharmacy, Princess Nourah Bint Abdulrahman University, Riyadh 11671, Saudi Arabia; 8 Department of Pharmaceutics, College of Pharmacy, University of Ha’il, Ha’il 81442, Saudi Arabia; 9 Department of Pharmaceutics and Industrial Pharmacy, Faculty of Pharmacy, Zagazig University, Zagazig 44519, Egypt; 10 Department of Pharmacology and Toxicology, College of Pharmacy, University of Ha’il, Ha’il 81442, Saudi Arabia

**Keywords:** anemia, iron, orodispersible film, plasticizer, pullulan

## Abstract

Iron deficiency is the principal cause of nutritional anemia and it constitutes a major health problem, especially during pregnancy. Despite the availability of various non-invasive traditional oral dosage forms such as tablets, capsules, and liquid preparations of iron, they are hard to consume for special populations such as pregnant women, pediatric, and geriatric patients with dysphagia and vomiting tendency. The objective of the present study was to develop and characterize pullulan-based iron-loaded orodispersible films (i-ODFs). Microparticles of iron were formulated by a microencapsulation technique, to mask the bitter taste of iron, and ODFs were fabricated by a modified solvent casting method. Morphological characteristics of the microparticles were identified by optical microscopy and the percentage of iron loading was evaluated by inductively coupled plasma optical emission spectroscopy (ICP-OES). The fabricated i-ODFs were evaluated for their morphology by scanning electron microscopy. Other parameters including thickness, folding endurance, tensile strength, weight variation, disintegration time, percentage moisture loss, surface pH, and in vivo animal safety were evaluated. Lastly, stability studies were carried out at a temperature of 25 °C/60% RH. The results of the study confirmed that pullulan-based i-ODFs had good physicochemical properties, excellent disintegration time, and optimal stability at specified storage conditions. Most importantly, the i-ODFs were free from irritation when administered to the tongue as confirmed by the hamster cheek pouch model and surface pH determination. Collectively, the present study suggests that the film-forming agent, pullulan, could be successfully employed on a lab scale to formulate orodispersible films of iron. In addition, i-ODFs can be processed easily on a large scale for commercial use.

## 1. Introduction

Iron (Fe) is a key micronutrient essential for various biochemical functions in the human body. Its deficiency impacts oxygen transportation and enzymatic reactions involved in various metabolic pathways [1]. Progressive augmentation in iron deficiency would lead to a medical condition called ‘Anemia’ or more specifically ‘Iron Deficiency Anemia (IDA)’ [2]. Subjects such as pregnant women and children are highly vulnerable groups to IDA. This medical condition is determined based on the percentage of hemoglobin (Hb) present in red blood cells. As per the World Health Organization, adult males are said to be anemic if the Hb value is <13.0 g/dL. Similarly, females and pregnant women are called anemic if the Hb values are <12.0 g/dL and <11.0 g/dL, respectively [3]. Various strategies exist to counter IDA that include but are not limited to iron supplementation or iron food fortification using various salts of iron [4]. Clinically, various non-invasive traditional oral dosage forms (tablets, capsules, and liquid preparations) of iron are commonly used as the first line of therapy [5]. Nevertheless, they are hard to consume for special populations such as pregnant women, pediatric, and geriatric patients with dysphagia, vomiting tendency, bipolar disorder, oral cancer, and Parkinson’s disease [6,7,8,9]. Moreover, these dosage forms also cause dose-dependent side effects such as gastric upset, diarrhea, nausea, metallic taste [10], and changes in the gut microbiome [11,12,13]. At the same time, the parenteral administration of iron is associated with various disadvantages. For instance, intramuscular administration leads to local reactions such as pain, skin staining (brown), atrophy, necrosis, and immediate reaction like anaphylactic shock. Similarly, intravenous administration of iron may lead to phlebitis and various systemic reactions such as fever, dizziness, myalgia, arthralgia, and vomiting [14]. Nonetheless, in very severe IDA (Hb < 8.0 g/dL), parenteral iron therapy is preferred due to its superior rapidity and efficacy [15]. 

The above-mentioned limitations in general and the difficulties of the special population, in particular, highlight the need for a novel drug delivery system. Accordingly, the pharmaceutical and clinical research industries have witnessed a transition in oral drug delivery from traditional dosage forms (tablets or injections) to a new non-invasive concept of rapidly dispersing orodispersible films (ODFs) [16,17]. ODFs are ultra-thin, portable, and postage-stamp-sized dosage forms that ‘rapidly disperse when administered to the tongue and swallowed naturally along with the saliva to get absorbed into the systemic circulation via the gastrointestinal tract’ [17]. Most importantly, compared to traditional dosage forms, ODFs do not need water for administration and are easy to administer to patients across all age groups [18]. Thus, they are especially well-positioned to meet the requirements of the special population (pediatrics geriatrics, and pregnant women) suffering from dysphagia, nausea/vomiting, and other conditions or diseases stated above [6,7,9]. 

Various film-forming polymers are employed in the fabrication of ODFs, such as maltodextrin [19], polyvinyl alcohol [20], hydroxypropyl methylcellulose [21], and pullulan [22]. These polymers offer tremendous support in maintaining the film’s mechanical properties, disintegration and dissolution, and good quality with an acceptable mouthfeel. Of particular interest, pullulan is a natural, biocompatible, biodegradable, non-ionic, water-soluble material obtained from the black yeast *Aureobasidium pullulan* [23,24]. Pullulan has been used in film formulations of various drugs and has been found to be a safe and non-toxic material [25,26]. Accordingly, in this study pullulan was employed as a polymer in the formulation of i-ODFs. 

Generally, the most commonly used source of iron supplements for therapeutic and prophylactic use is ferrous sulfate, which is freely soluble in water [27,28]. However, its use is associated with common side effects such as the darkening of teeth, nausea, abdominal pain, and black/ dark stools [29,30,31,32]. To overcome these issues, compounds such as ferrous succinate, ferrous fumarate, and ferric saccharate have evolved, which are poorly soluble in water but soluble in dilute hydrochloric acid, have less organoleptic issues compared to ferrous sulfate, and readily enter the common iron pool during digestion [33]. Nevertheless, these salts are not bioavailable and need effective taste masking, since the acceptability of any ODF depends predominantly on its taste. Hence, the taste of the ODF is a crucial factor to gain end-user acceptance and thereby compliance. Therefore, effective strategies to taste-mask the active substance need to be developed to formulate an ideal ODF [34]. 

Accordingly, in light of the above rationale and background of iron and pullulan, the objective of the present study was to develop a palatable, rapidly dispersing, non-irritant, and readily bioavailable pullulan-based iron-loaded ODF (i-ODF) for prophylactic use in subjects across all age groups and especially for people with special needs. 

## 2. Materials and Methods

### 2.1. Materials

Ferric saccharate was obtained from Biodeal Pharmaceuticals (New Delhi, India). The film-forming polymer, pullulan, was purchased from Kumar Organics (Bangalore, India). Sodium alginate, calcium acetate, and all other pharmaceutically acceptable excipients employed in the formulation of the ODFs were purchased from S.D. Fine Chemicals Ltd. (Mumbai, India). All other chemicals and solvents used were of analytical grade. 

### 2.2. Preparation of Microencapsulated Iron Particles

Table 1 provides details of the composition to fabricate microencapsulated or microparticles (MPs) of iron (Fe). The fabrication steps include dissolving sodium alginate and ferric saccharate in 100 mL of distilled water to obtain a viscous mixture. The mixture obtained was added dropwise under controlled magnetic stirring (at a temperature of 25 °C) to a solution of calcium acetate to obtain MPs of Fe [35,36]. The obtained particles were separated by filtration under vacuum and resuspended in distilled water to remove soluble salts, if any. This step was repeated to remove all the soluble salts. Finally, the particles were filtered under a vacuum to obtain Fe MPs. Thereafter, the MPs were dried at room temperature and subjected to further characterization studies or stored in a well-closed container with a small amount of talc till further use. 

### 2.3. Characterization of the Iron Microparticles

#### 2.3.1. Optical Microscopy

The size of the Fe MPs was estimated using an optical microscope (Nikon Eclipse, E800, Tokyo, Japan). A small portion of the MPs (aggregate) along with a drop of water was placed on a slide and covered with a coverslip for microscopic examination using an optical microscope. The size was measured using a calibrated and superimposed scale on each image. 

#### 2.3.2. Inductively Coupled Plasma Optical Emission Spectroscopy

The amount of calcium and iron in the Fe MPs was estimated using inductively coupled plasma optical emission spectroscopy (ICP-OES) (iCAP Pro, ICP-OES, Thermo Fisher Scientific, Waltham, MA, USA). A small portion (200 mg) of the MPs of Fe was combined with nitric acid and digested in a microwave oven and subjected to ICP-OES analysis. 

#### 2.3.3. Scanning Electron Microscopy (SEM) with X-ray Energy Dispersion (EDS)

A high-resolution SEM (Carl Zeiss, Neon Crossbeam, Berlin, Germany) equipped with a spectrophotometer with an EDS (EDAX company, Unterschleissheim, Germany) was employed for the study. A voltage of 5 to 10 kV was employed as an acceleration voltage to obtain SEM images of the Fe MPs. A semi-quantitative detection was performed to detect the elemental composition of Fe MPs using an acceleration voltage of 10 kV. 

### 2.4. Fabrication of Pullulan-Based ODFs Loaded with Fe MPs

A solvent casting method with minor modifications was employed to fabricate iron ODFs. Table 2 lists the ingredients and quantities employed in the preparation. First, the film-forming polymer, pullulan, was dissolved in water and left overnight to obtain a clear solution. Similarly, lecithin was dissolved separately in a portion of the solvent. Fe MPs and beta-cyclodextrin were mixed in water under continuous stirring followed by the addition of mannitol and sweetening agents. Other ingredients, namely, calcium carboxymethylcellulose, ascorbic acid, and malic acid, were also added under continuous stirring for 15 min. Other excipients, namely, polyethylene glycol, sorbitol, and flavoring agents were then added under continuous stirring. Thereafter, the obtained solution along with the lecithin solution was added to the solution of pullulan under continuous stirring for about 20 min to obtain a homogenous slurry and subjected to deaeration under vacuum (pressure between 600 to 700 mm of Hg) for 2 to 3 h to remove air bubbles, if any. Finally, the solution was cast as a layer using an automatic layering machine with a predetermined thickness. Thereafter, the film is removed carefully and dried at a temperature of 60 °C followed by cutting and packaging in a tri-laminate aluminum pouch. The packed films are stored in a desiccator to prevent moisture or microbial attack and were used for further characterization.

### 2.5. Characterization of the ODFs

#### 2.5.1. Physical Examination

The fabricated pullulan-based i-ODFs were physically examined with respect to their appearance (using the naked eye), handling property, and texture. 

#### 2.5.2. Weight

The ODF samples were cut into a size of 2 × 2 cm^2^ and weighed on an electronic balance (Mettler-Toledo, Mumbai, India). The average weight was considered as a mean weight variation. 

#### 2.5.3. Thickness

The thickness of the film was measured using a screw gauge having an accuracy of 0.001 mm. Measurements were taken from the center and also from the four corners of the film having the size of 2 × 2 cm^2^. The thickness measured is reported as mean ± SD. 

#### 2.5.4. Drug Content

To ensure the drug payload in the film, drug content analysis was done. A premeasured region of the film was dissolved in phosphate buffer (50 mL) by stirring followed by filtration through a filter paper (0.45 µm) and the amount of drug was determined by high-performance liquid chromatography (HPLC) method. The method employed a mobile phase comprising phosphate buffer (pH 2.5) and methanol in a ratio of 970:30 (flow rate: 1.0 mL/min). The detection wavelength was 210 nm and the run time was 15 min. 

#### 2.5.5. Disintegration Time

The disintegration time of the ODFs was measured by a Petri dish method [37], wherein a film of 2 × 2 cm^2^ was placed in a Petri dish having 10 mL of phosphate buffer having pH of 6.8 (simulated salivary fluid; Table S1). The time taken for the ODF to disintegrate completely was measured using a stopwatch and the same experiment was repeated thrice and the mean value is reported. 

#### 2.5.6. Folding Endurance

The fabricated ODF was folded repeatedly at a predetermined spot until it broke. The number of times the film can be folded without breaking is taken as the value of folding endurance. The value was measured in triplicate.

#### 2.5.7. Tensile Strength

To measure tensile strength, the ODF having a size of 2 × 2 cm^2^ was clamped at one end and the other end was attached to a hanging pan for loading weights. The total weight required for the breakage of the film was estimated. The experiment was performed in triplicate and the tensile strength is reported as an average.

$$Tensile\;Strength=\frac{Force\;at\;break\;(N)}{Initial\;area\;of\;the\;film\;({cm}^{2})}$$

#### 2.5.8. Surface pH

An ODF sample was moistened with distilled water (0.5 mL) and left for 2 min. The pH of the moistened film was measured using a pH meter (IKON Instruments, Delhi, India). In this method, the surface of the pH meter electrode was touched with the moistened surface of the film to measure the pH. Readings were taken in triplicate for each sample and the average value of the readings is reported. 

#### 2.5.9. Karl–Fischer Titration (Water Content)

To determine the water content, Karl–Fisher titration was performed using a DL37 coulometric titrator (Mettler-Toledo, Mumbai, India). One ODF sample (2 × 2 cm^2^) was added to about 5 mL methanol and the titration was continued until it reaches an electrometric endpoint. The water content determination was performed in triplicate corrected for solvent water content. The moisture content was calculated using the formula: 

#### 2.5.10. Morphology by Scanning Electron Microscopy (SEM)

The overall surface morphology of ODFs loaded with Fe MPs was evaluated by scanning electron microscopy (SEM) (Carl Zeiss, Neon Crossbeam, Berlin, Germany). The ODF sample (size: 1 mm^2^) was positioned on a circular aluminum stub and sputter coated with Au/Pd under argon atmosphere using a vacuum evaporator followed by scanning using SEM. 

#### 2.5.11. Fourier Transform Infrared Spectroscopy (FTIR)

FTIR spectra (scanned between 4000 and 400 cm^−1^; resolution: 4 cm^−1^ for 20 scans) of the film-forming material, pullulan, ferric saccharate, iron-loaded microparticles, and the physical mixture for ODFs consisting of the active substance (iron) was carried out. The spectra were obtained by the traditional potassium bromide disc method followed by FTIR analysis (Shimadzu, model 8400, Tokyo, Japan) [38]. 

#### 2.5.12. Microbial Load

The ODF samples were tested for microbial burden (aerobic colony count, molds, yeasts, and coliforms) as per ISO 11737-1 for determining the microbial load (population) in a product. 

### 2.6. Dissolution Studies

An in vitro dissolution study of pullulan-based i-ODFs was performed in triplicate using a USP XXXIV paddle apparatus (type II) with 900 mL of 0.1 N HCl as dissolution media at 50 rpm and 37 ± 0.5 °C. Samples (aliquots of 5 mL) were collected periodically and replenished with equivalent volumes of fresh dissolution medium to maintain sink conditions. The collected samples were filtered through a filter paper (0.45 µm) and the amount of drug released was determined by high-performance liquid chromatography (HPLC) as aforementioned. 

### 2.7. Stability Studies

The optimized ODF formulation was subjected to stability studies at a temperature of 25 ± 2 °C and relative humidity of 60% ± 5% for 90 days. The ODFs were packed in a tri-laminate aluminum pouch and kept in a stability chamber. After the stipulated periods of 30, 60, and 90 days, the i-ODF samples were analyzed for their appearance (physical), thickness, drug content, folding endurance, surface pH, disintegration time (seconds), and dissolution profile. 

### 2.8. In Vivo Biocompatibility Study Using Hamster Cheek Pouch Model (Irritation Study)

The mucosal irritation caused, if any, by i-ODF was assessed using a hamster cheek pouch model. This model was employed to study the safety of i-ODFs in the oral cavity. Hamsters of each sex were housed individually and provided with controlled environmental conditions of 12 h light/dark cycle with free access to both food and water. After acclimatizing for seven days, hamsters weighing between 100 to 150 g were selected for the study. The complete experimental procedures were carried out as per The Committee for the Purpose of Control and Supervision of Experiments on Animals (CPCSEA) guidelines. The entire study was reviewed and approved by the Institutional Animal Ethics Committee (IAEC/JSSCPM/349/2020), Mysuru, Karnataka, India. 

Study design: Hamsters were divided into two groups; Group I and Group II. Group I was kept on placebo film while Group II was kept on i-ODFs. Respective films were administered (positioned in the hamster’s cheek pouch) for a time period of about 10 min followed by rinsing with distilled water. Thereafter, the pouch was immediately observed for redness (irritation), if any, and also after 24 h period. The placebo and i-ODFs were administered to hamsters twice a day for a time period of 4 to 5 days. 

## 3. Results and Discussion

### 3.1. Microencapsulated Iron Particles and Their Characterization

**Figure 1:**
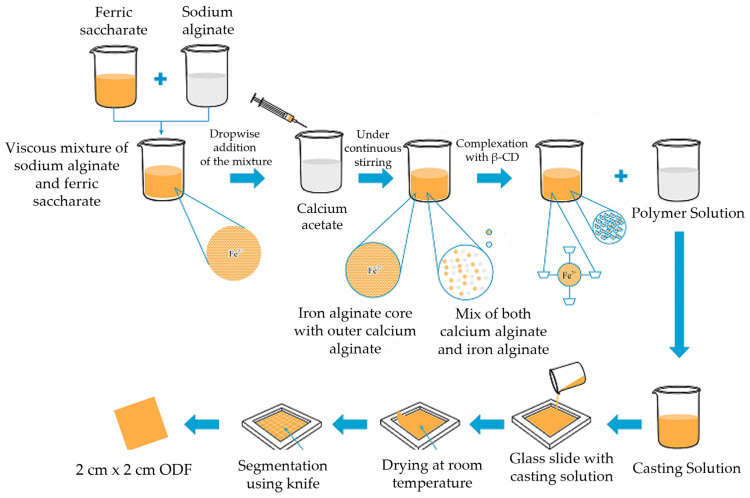
Schematic representation of preparation of iron-containing orodispersible films.

Microencapsulation is a popular technology, wherein the active moiety is incorporated within a polymeric shell to prevent its interaction with the outside environment (other agents or oral mucosa) till it reaches the intestine. It also helps reduce the metallic taste in the oral cavity. Further, this technology offers protection against oxidation or heat, and thereby, enhances the shelf-life of the product. Sodium alginate is a natural polysaccharide consisting of β-D-mannuronic acid and α-l-glucuronic acid. When dissolved in water, it tends to form reticulated structures, wherein the anionic acid groups react with cations (divalent or polyvalent) to form an insoluble network. In the synthesis of Fe MPs, the viscous mixture of ferric saccharate and sodium alginate when introduced into the solution of calcium acetate forms an insoluble network by ionic cross-linking of the alginate, wherein the glucuronic acid portion of the alginate molecules forms junctions with calcium molecules, popularly called as egg-box [39]. Following this, the mixture was combined with β-cyclodextrin to undergo complexation. The host-guest interactions or linking between cyclodextrin with microencapsulated iron particles (Figure 1) help obtain a mixture that is ready for mixing with the solution of pullulan, the film-forming material.

### 3.2. Characterization of Iron Microparticles

Macroscopic aggregates were observed by the naked eye and these were very evident when seen under the microscope. The size of the aggregates ranged between 0.1 and 1 mm. Nonetheless, the size of the Fe MPs of Fe ranged from 5 to 15 µm, which is acceptable to proceed with the next steps of the fabrication process [40]. The amount of iron and calcium in batches P1 and P2 are expressed as a percentage of weight as shown in Table 3. From the results, it is evident that good iron loading capacity was observed in both batches. 

**Figure 2:**
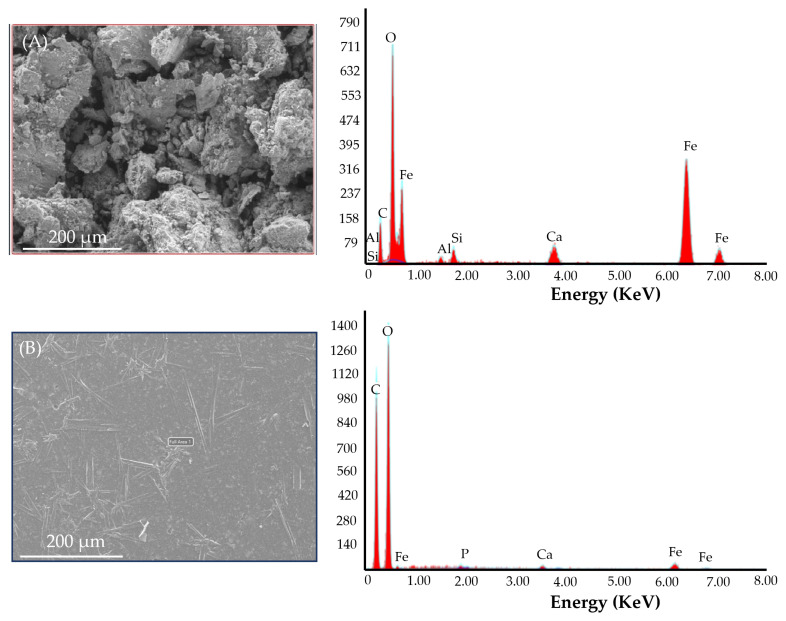
EDS analysis of (**A**) microparticles of iron and (**B**) pullulan-based iron-loaded orodispersible films.

Additionally, EDS analysis was performed on the Fe MPs and i-ODFs. The SEM images and EDS graph are represented in Figure 2. In addition, the weight percentages of different elements present in the Fe MPs and i-ODFs are summarized in Table 4. As depicted in Table 4, significant differences in the weight percentages of carbon and oxygen were observed between the Fe MPs and i-ODFs. Furthermore, the weight percentage of Fe in the microparticles was significantly higher than that in the orodispersible films. This is probably due to the complexation of Fe with β-CD in the film formulation. Other elements, namely, silicon and aluminum, are present in the Fe MPs but are absent in the film formulation.

### 3.3. Iron Orodispersible Films (i-ODFs)

#### Selection of Excipients

**Figure 3:**
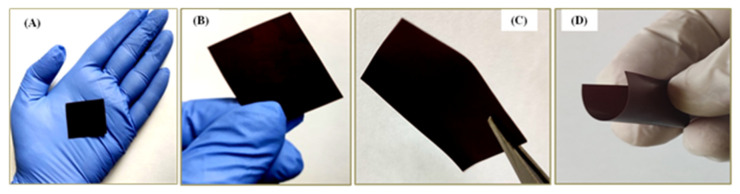
Pullulan-based iron-loaded orodispersible films (i-ODFs). (**A**,**B**) optimized formulation (F6) fabricated by modified solvent casting method; (**C**) side view of i-ODF showing thickness of the film; and (**D**) flexibility of the i-ODF.

The oral delivery of drugs or nutrients using ODF technology is a relatively novel and inventive method. The solvent casting method stands out as the most preferred and industrially acceptable method for fabricating ODFs [8]. Different formulation batches (F1 to F6) were fabricated as summarized in Table 2. Glycerol, PEG 600, and sorbitol 70% were employed as plasticizers to help maintain the mechanical properties of the film. Lecithin and Tween 80 act as emulsifying agents to reduce the surface tension between the oily flavoring agents (such as kiwi and grape flavor) with the aqueous portion of the formulation. Some of the trial batches had problems such as the peeling of the film from the glass surface. To alleviate this issue, mannitol (5%) was employed as an anti-caking agent and helped in the smooth peeling of the film after casting from the glass surface. Ascorbic acid helps in enhancing the absorption of iron and malic acid helps stimulate the secretion of saliva in the oral cavity when the ODF is administered to the tongue. Calcium carboxymethyl cellulose is used as a disintegrating agent to facilitate rapid disintegration of the ODF when administered to the tongue. Different sweetening agents were employed in the formulation, namely, glucose, fructose, steviose 100, stevirome 5000, and steviol glycosides. The essential nutrients and pharmaceutically acceptable and compatible excipients used in the present study are listed in the United States Food and Drug Administration’s list of Generally Recognized as Safe (GRAS) substances. The final optimized formulation (Figure 3) was subjected to further characterization to study the formulation properties and its stability.

### 3.4. Characterization of the Pullulan-Based Iron-Loaded Orodispersible Films

#### 3.4.1. Acceptability by the Subjects

As a quality criterion for acceptability by the subjects, the ODF must have a smooth texture and be free from air bubbles, flexible and soft to ensure excellent mouthfeel and good handling to gain patient acceptability and thereby compliance [41]. The ODFs of the present study when physically examined were found to be dark brown, free from air bubbles, flexible, and smooth textured, with moderate thickness. Formulation batches F5 and F6 were flexible, smooth, and thin, and thus were judged to be associated with good handling properties. Accordingly, they were assumed to possess good mouthfeel and thereby help in avoiding compliance issues from the subjects. Parameters such as disintegration time, mechanical properties, and moisture content were considered to be critical quality attributes that might affect the performance of the ODF [42,43]. Generally, ODFs should be easily portable (handling and transportation), rapidly disintegrate when administered to the tongue, and should have an allowable moisture limit to facilitate flexibility and must avoid tackiness. High moisture content can directly impact product stability (microbial and chemical) [41]. The results of desired evaluation parameters of different batches (F1 to F6) are shown in Table 5. 

#### 3.4.2. Weight Variation

The results obtained after measuring the weight are shown in Table 5. All the formulation batches exhibited narrow weight distributions with a low coefficient of variation. This parameter indicates uniformity in the distribution of the active essential nutrient, iron, in the pullulan-based ODF, as well as excipients. The weight of the pullulan-based iron ODFs ranged from 145 to 155 mg. The mean ± standard deviation weight of the 4 cm^2^ ODF was 150 ± 5 mg, thereby signifying uniformity in the ODFs. 

#### 3.4.3. Thickness

Thickness is a physical property of the ODF that warrants uniformity in the casting process. The thickness results are tabulated in Table 5. The ODFs must neither be too thin to avoid handling issues nor too thick as it would have a direct impact on the disintegration time of the film. The optimized formulation batch (F6) exhibited an average thickness of 0.15 ± 0.01 mm. A small standard deviation indicates the uniformity in the ODFs and, therefore, the absence of drug islands or islands of undissolved substances in the film. 

#### 3.4.4. Folding Endurance (FE)

Folding endurance is a qualitative parameter that helps measure the mechanical strength such as brittleness or flexibility of the ODF. High FE indicates higher mechanical strength. The optimized formulation batches showed FE values ranging between 100 to 120 folds, indicating that the pullulan-based i-ODFs would be mechanically strong (not brittle in nature) during handling and while being administered to the tongue. In addition, the incorporation of the plasticizer combination (PEG 600 and sorbitol 70%) significantly enhanced the FE of pullulan-based i-ODFs (F6). Collectively, the film-forming polymer, pullulan, offered enough strength to the film and the plasticizer combination (PEG 600 and sorbitol 70% in a ratio of 2:4) exhibited flexibility in the optimized formulation batch (F6).

#### 3.4.5. Surface pH

The surface pH of the pullulan-based iron ODFs ranged from 6.5 to 6.8, which was within acceptable limits. The ideal pH value must be close to 7.0 to avoid mucosal irritation in the oral cavity, thereby, helping in gaining patient compliance. 

#### 3.4.6. Disintegration Time

**Figure 4:**
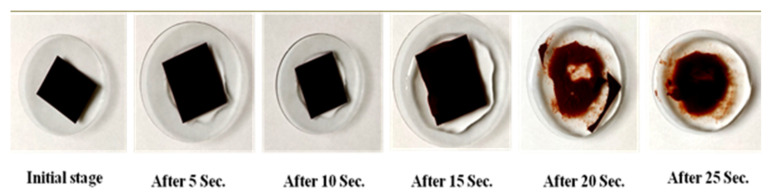
In vitro disintegration time of pullulan-based iron-loaded orodispersible films (i-ODFs) of batch F6 in phosphate buffer with a pH of 6.8.

The Petri-dish method was adopted to determine the disintegration time of the ODF. The disintegration time of the pullulan-based iron i-ODFs in phosphate buffer (pH 6.8) was found to be below 25 s, thereby indicating rapid disintegration of the films. The complete disappearance of the disintegrated film into solution happened within 40 s. This is further indicated by the brown-colored solution (Figure 4).

#### 3.4.7. Dissolution Profile

The iron-loaded ODFs administered to the tongue undergo rapid disintegration due to salivary hydration and are naturally swallowed along with saliva into the stomach via the esophagus. Absorption of iron occurs predominantly in the duodenum and upper jejunum. A variety of factors influence the absorption of iron, of which vitamin C (ascorbic acid) enhances iron uptake. In addition, the physical state of the iron entering the duodenum also plays a pivotal role in its absorption. Quick release of iron is vital for its absorption in the duodenum and upper jejunum. All the formulation batches showed a rapid release of iron from pullulan-based ODF (Table 5). It was observed that almost all batches showed Q5min of more than 90%. However, a few batches (F1 and F2) showed a Q5min of more than 85% but less than 90% due to a different plasticizer (glycerol + glycerol oleate) combination than the other batches (Figure S1). Therefore, from the dissolution study, it was evident that the combination of plasticizers (sorbitol 70% + PEG 600) with pullulan shows a better release profile. 

#### 3.4.8. FTIR Spectrophotometric Method

**Figure 5:**
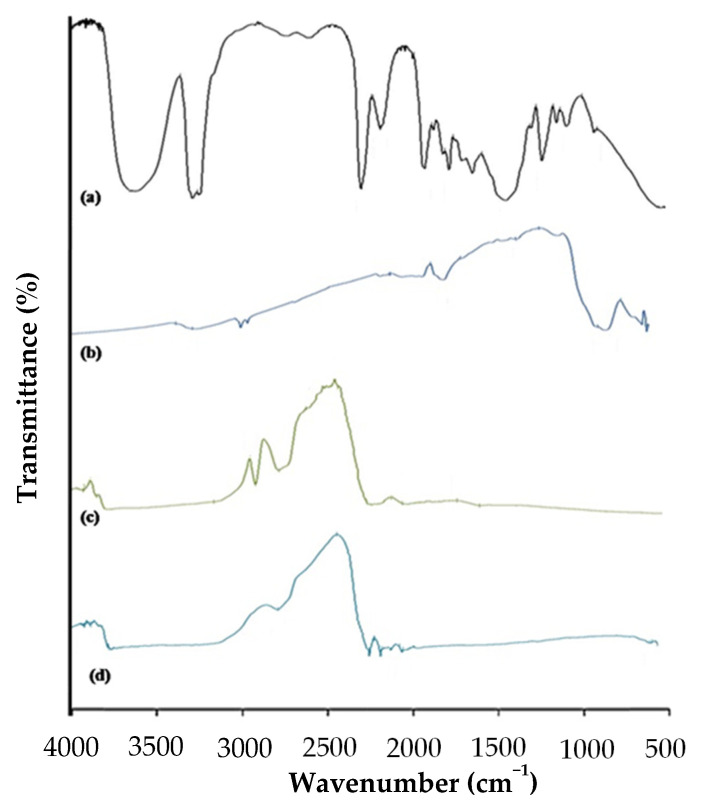
Comparison of FTIR spectra of (**a**) pullulan, (**b**) ferric saccharate, (**c**) physical mixture of iron-loaded microparticles, and (**d**) iron-loaded orodispersible film (i-ODF).

FTIR spectra of pure pullulan, ferric saccharate, the physical mixture of iron-loaded microparticles, and the final i-ODF are shown in Figure 5. In the spectra of pure pullulan, a sharp peak due to C=O stretching of both keto groups was observed at 1732 cm^−1^. Similarly, in the spectra of ferric saccharate absorption bands are seen in the fingerprint region at 1033 cm^−1^ due to C-O-C stretching vibration, at 1691 cm^−1^ due to C=O five-membered cyclic stretching, and at 3427 cm^−1^ due to aromatic N-H stretching vibration. Conversely, broad peaks were observed for the physical mixture of ferric saccharate at 1811 to 1957 cm^−1^ and for the i-ODF between 1678 and 2158 cm^−1^. The comparative spectra (Figure 5) clearly illustrate the absence of chemical interaction between pullulan and the excipients.

#### 3.4.9. Morphological Study Using SEM 

**Figure 6:**
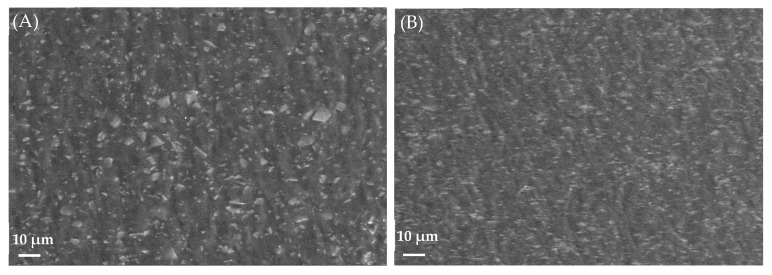
Micrographs by scanning electron microscopy (SEM) (**A**) images of sample A with rough surfaces and (**B**) images of sample B with smooth surfaces.

SEM studies were performed to examine the uniformity of the dispersion of ingredients in the pullulan-based i-ODFs. The analysis was carried out for sample A (iron-loaded pullulan-based ODFs without sweetening agents, and grape flavor) and sample B (iron-loaded pullulan-based ODFs with all the ingredients). The SEM images (Figure 6) show the difference between samples A and B. The surface of the ODF of sample A was rough/coarse when compared with the surface of sample B. This difference is primarily due to non-homogeneity in the dispersion of solids in the film-forming solution, pullulan, before casting the ODF. Accordingly, it can be inferred that good homogenization of all the ingredients is vital before casting the film to obtain an ODF with a smooth surface after the casting and drying steps. Most importantly, the SEM images (Figure 6) of the pullulan-based i-ODFs revealed good uniformity in the ODF. No aggregates were observed in the ODF, which could have formed during the drying process.

#### 3.4.10. Tensile Strength

Tensile strength is another parameter used to characterize the mechanical properties of the ODF. It indicates how robust the ODF is to withstand stress during its journey from fabrication to handling by the end-user. In addition, a too-rigid film could cause a bad mouthfeel. A tensile strength of 165 ± 0.35 g/cm^2^ was found to be optimum enough to offer the required toughness to the ODF to handle the stress experienced from fabrication to administration by the subject. 

#### 3.4.11. Karl–Fischer Titration

The Karl–Fischer titration method helps determine the water content, if any, present in the pullulan-based i-ODF samples. Borges and colleagues recommended residual water content ranging between 3 to 6% *w/w* as ideal for the stability of an ODF [35]. The water content of the final formulation was found to range from 4.0 to 5.0% *w/w*. Therefore, this value indicates good physical stability and integrity of the ODF. 

#### 3.4.12. Microbial Bioburden

The microencapsulated iron ODF samples were tested for the presence of microorganisms by in-house standard testing procedures. The test procedure involves testing the samples for the presence of microorganisms, namely, yeast and molds, *E. coli* (bacteria), *Staphylococcus aureus*, and *Pseudomonas aeruginosa*. Except for yeast and molds (with <10 CFU/g), the remaining microorganisms were absent. Accordingly, the formulated ODFs were found to be within the pharmaceutically acceptable criteria (total aerobic count: <10^3^ CFU/g and total yeast and molds <10^2^ CFU/g) from the microbiological point of view for non-sterile dosage forms [44]. 

#### 3.4.13. Drug Content

The iron content in the sample ODF, as determined by HPLC, was found to be 99.4% of the label claim (27 mg per each strip of ODF) as the average value for the optimized formulation. 

### 3.5. Stability Studies

**Figure 7:**
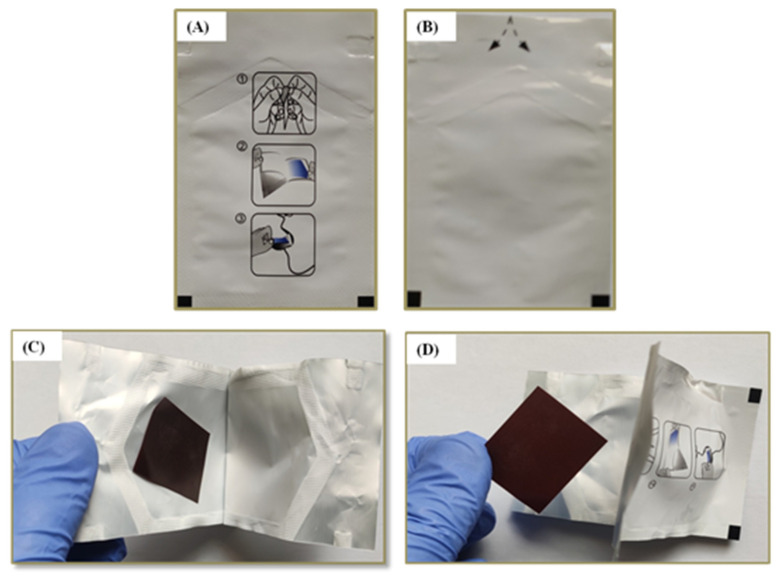
Tri-laminate aluminum pouch for packaging iron orodispersible films (i-ODFs): (**A**) Front view of the pouch with pictorial instructions to open and consume i-ODF and (**B**) Back view showing directions to open (**C**) single pullulan-based i-ODF per pouch in an open position (**D**) i-ODF removed after stability studies.

Stability studies were performed for the optimized i-ODF formulation (F6) by investigating the visual appearance, thickness, drug content, folding endurance, surface pH, disintegration time (seconds), and dissolution profile after a period of 30, 60, and 90 days, and the results are summarized in Table 6. The study results confirmed a lack of any significant variation in the desired properties of the film formulation such as surface thickness, pH, disintegration time, appearance, folding endurance, and drug content over three months when packed and stored in the tri-laminate aluminum pouch (Figure 7) at 25 ± 2 °C and a relative humidity of 60% ± 5%. Most importantly, the iron content in the ODF after 90 days was found to be 98.51 ± 0.45%. Accordingly, the pullulan-based i-ODFs were found to be stable for 90 days when stored in a tri-laminate aluminum pouch. Therefore, the tri-laminate aluminum pouch is considered ideal for packaging and storing pullulan-based i-ODFs. 

### 3.6. In Vivo Biocompatibility Study Using Hamster Cheek Pouch Model (Irritation Study)

Generally, the formulated i-ODFs when administered to the tongue tend to come in contact with oral mucosa. Therefore, biocompatibility studies of i-ODFs are a prerequisite to clinical use. The hamster cheek pouch model [9] was found to be an appropriate model to test the irritation of i-ODFs, if any, when administered via the oral route. Based on macroscopic observations, no redness (irritation) was observed on the cheek pouch area of hamsters both before and after placing i-ODFs. Accordingly, the fabricated pullulan-based i-ODFs were deemed free from irritation. 

## 4. Conclusions

Taste-masked pullulan-based i-ODFs were successfully fabricated by a solvent-casting method. The optimized batch (F6) exhibited excellent disintegration time, good folding endurance, a high percentage of drug release, and good stability when stored in a tri-laminate aluminum pouch. In addition, the incorporation of mannitol in all the formulation batches helped in acting as an anti-caking agent and facilitated the peeling of the film from the smooth casting surface, which in turn, might enhance acceptability by the subjects. Most importantly, the film is free from irritation when administered to the tongue as confirmed by a hamster cheek pouch model and surface pH determination (pH = 6.5 to 6.8). In summary, the film-forming agent, pullulan, was successfully employed on a lab scale to formulate orodispersible films of iron. Furthermore, the ODFs of the present study can be processed easily on a large scale and can be commercialized successfully. 

## Figures and Tables

**Table 1:** Composition of microencapsulated iron particles.

Batch No.	Ferric Saccharate (% *w/v*)	Sodium Alginate (% *w/v*)	Calcium Acetate (M)
P1	35	1.5	0.1
P2	35	3.0	1.0

**Table 2:** List of ingredients used for the preparation of iron orodispersible films (i-ODFs).

Ingredients	Composition% *w/w*					
	F1	F2	F3	F4	F5	F6
Microencapsulated iron	30.833	28.462	29.600	27.407	25.517	24.667
Pullulan	35.833	36.154	37.600	34.815	32.414	29.333
Beta cyclodextrin	-	-	-	-	13.793	13.333
Zinc lactate	-	3.846	-	-	-	-
DCP (Dicalcium Phosphate)	4.167	3.846	-	-	-	-
Mannitol	4.267	3.131	4.136	2.348	3.241	3.533
Glucose	4.167	3.846	4.000	3.704	3.448	3.333
Fructose	4.167	0.615	4.000	3.704	3.448	3.333
Steviose 100	-	-	-	-	-	0.533
Stevirome 5000	-	-	-	-	-	1.200
Steviol glycosides	0.667	3.846	0.640	0.593	0.828	-
Calcium CMC	4.167	0.769	4.000	3.704	3.448	3.333
PEG 600	-	-	-	-	-	1.333
Sorbitol 70%	-	-	-	-	-	2.667
Glycerol Triacetate	-	0.769	0.800	0.741	1.379	-
Tween 80	0.833	-	-	-	-	-
Lecithin	1.500	0.769	1.440	1.333	1.379	2.667
ascorbic acid	0.125	0.769	0.080	0.074	0.069	0.067
Malic acid	-	3.077	3.200	2.963	2.759	2.667
Glycerol	4.167	3.846	4.000	3.704	2.759	-
Glycerol oleate	0.108	0.100	0.104	0.096	-	-
Grape flavor	5.000	6.154	6.400	5.926	-	-
Kiwi flavor	-	-	-	8.889	5.517	8.000

**Table 3:** Concentration of iron and calcium in batches P1 and P2.

Item	Batches	
	P1	P2
% Fe	7.5	8.0
% Ca	0.5	1.1

**Table 4:** Weight percentages of different elements in iron microparticles and pullulan-based orodispersible films of iron.

Name of the Element	Iron Microparticles (%)	Pullulan-Based Iron Orodispersible Film (%)
**C**	11.5	39.5
**O**	19.1	53.2
**P**	-	0.5
**Ca**	6.8	1.0
**Fe**	60.2	5.8
**Al**	1.0	-
**Si**	1.4	-
**Total**	100	100

**Table 5:** Results of desired evaluation parameters of batches F1 to F6.

Parameter	F1	F2	F3	F4	F5	F6
Weight variation (mg)	145 ± 4.0	145 ± 5.0	140 ± 5.0	150 ± 4.0	150 ± 5.0	150 ± 3.0
Thickness (mm)	0.13 ± 0.01	0.14 ± 0.01	0.14 ± 0.01	0.13 ± 0.01	0.15 ± 0.01	0.15 ± 0.01
Folding endurance	100 ± 5.0	115 ± 5.0	110 ± 5.0	105 ± 5.0	100 ± 5.0	120 ± 5.0
Surface pH	6.5 ± 0.3	6.4 ± 0.3	6.6 ± 0.2	6.3 ± 0.2	6.4 ± 0.2	6.7 ± 0.2
Disintegration time (s)	41 ± 3.0	41 ± 2.0	40 ± 5.0	40 ± 3.0	25 ± 1.0	23 ± 2.0
Tensile strength (g/cm^2^)	162 ± 0.31	160 ± 0.74	159 ± 0.11	167 ± 0.74	163 ± 0.16	165 ± 0.35
Dissolution release profile	86.50 ± 0.27	88.32 ± 0.53	90.25 ± 0.10	91.30 ± 0.17	95.17 ± 0.55	98.33 ± 0.35

All values shown here are mean ± S.D. (*n* = 3).

**Table 6:** Stability data of the optimized i-ODF formulation F6 (*n* = 3).

Parameter		Optimized Formulation (F6)		
	Fresh	30 Days	60 Days	90 Days
Drug content	99.4 ± 0.72	99.1 ± 0.54	98.7 ± 0.39	98.5 ± 0.45
Thickness (mm)	0.15 ± 0.01	0.1 5± 0.02	0.14 ± 0.01	0.14 ± 0.01
Folding endurance	120 ± 5.0	122 ± 3.0	124 ± 3.0	125 ± 2.0
Surface pH	6.7 ± 0.2	6.6± 0.1	6.5 ± 0.3	6.4 ± 0.5
Disintegration time (s)	23 ± 2.0	23 ± 2.0	24 ± 1.0	24 ± 3.0
Dissolution profile (%)	98.3 ± 0.35	98.5 ± 0.23	98.4 ± 0.42	98.4 ± 0.57

## Data Availability

Not applicable.

## Supplementary Materials

The following supporting information can be downloaded at: https://www.mdpi.com/article/10.3390/pharmaceutics15031027/s1, Figure S1: Dissolution rate of pullulan-based i-ODFs; Table S1: Composition of Simulated Salivary Fluid (SSF; pH 6.8).
