# Supplementary material for: Development and Characterization of Pullulan-Based Orodispersible Films of Iron
Source: Pharmaceutics. 2023 Mar 22;15(3):1027. doi: 10.3390/pharmaceutics15031027 (PMC10056616; doi:10.3390/pharmaceutics15031027)
Supplement: Supplementary file 1 [file pharmaceutics-15-01027-s001.zip › pharmaceutics-2236042-supplementary.pdf]

pharmaceutics

Supplementary Materials: Development and Characterization of Pullulan-Based Orodispersible Films of Iron

Maram Suresh Gupta, Tegginamath Pramod Kumar, Dinesh Reddy, Kamla Pathak, Devegowda Vishakante Gowda, A.V. Naresh Babu, Alhussain H. Aodah, El-Sayed Khafagy, Hadil Faris Alotaibi, Amr Selim Abu Lila, Afrasim Moin, Talib Hussin

Supplementary Information:

Table S1. Composition for simulated salivary fluid (SSF; pH 6.8).

Ingredients	Quantity
Disodium hydrogen phosphate	2.382 g
Potassium dihydrogen phosphate	0.19 g
Sodium chloride	8.00 g
Distilled water	Up to 1000 mL
Phosphoric acid	Q.S to pH 6.8

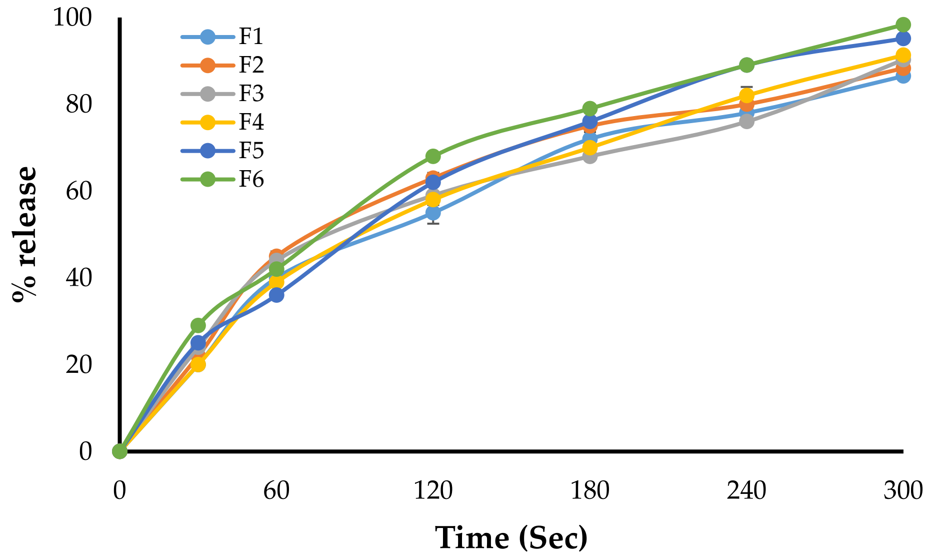

Figure S1. : Dissolution rate of pullulan-based i-ODFs.
